# Supplementary material for: Hypnotic enhancement of slow-wave sleep increases sleep-associated hormone secretion and reduces sympathetic predominance in healthy humans
Source: Commun Biol. 2022 Jul 26;5:747. doi: 10.1038/s42003-022-03643-y (PMC9325885; doi:10.1038/s42003-022-03643-y)
Supplement: Supplementary file 2 — Supplementary Information [file 42003_2022_3643_MOESM2_ESM.pdf]

Supplementary Figures

Hypnotic enhancement of slow-wave sleep increases sleep-associated hormone secretion and reduces sympathetic predominance in healthy humans

Luciana Besedovsky, Maren Cordi, Laura Wißlicen, Estefanía Martínez-Albert, Jan Born, Björn Rasch

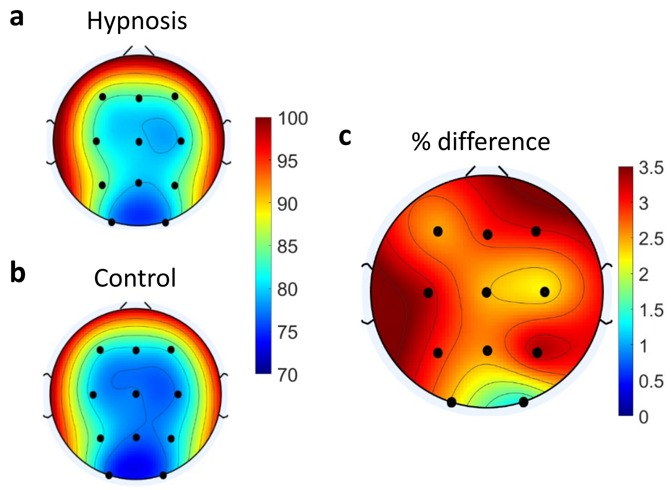

Supplementary Figure S1. Effect of hypnotic suggestions on slow-wave activity (SWA). Topographical distribution of SWA (0.5–4.5 Hz) during non-rapid eye movement (NREM) sleep after listening to (a) hypnotic suggestions or (b) a neutral control text. SWA is indicated as percent of total power. (c) Percent difference in SWA between the Hypnosis and the Control condition. Black dots represent electrode positions; n = 22.

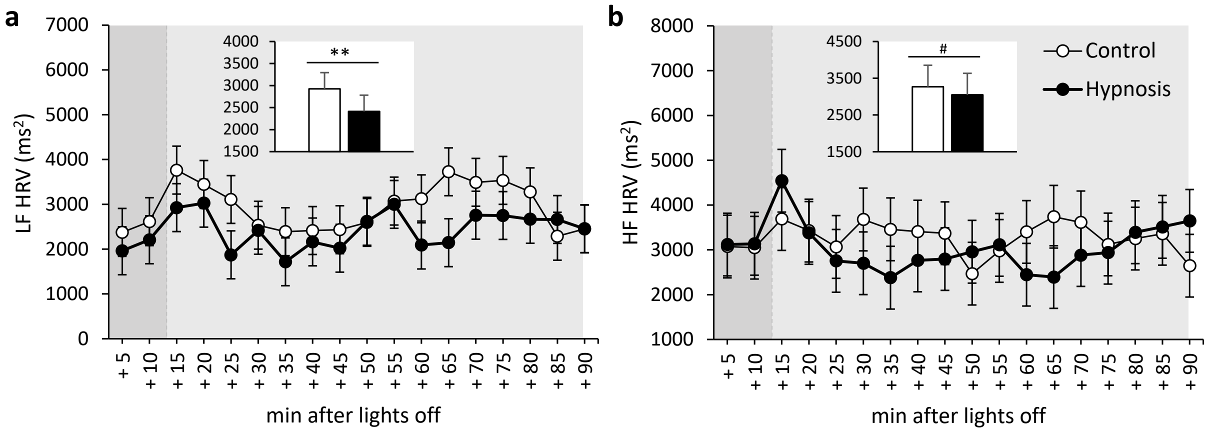

Supplementary Figure S2. Effects of hypnotic suggestions on measures of absolute low frequency (LF) and high frequency (HF) power of heart-rate variability (HRV). Estimated marginal means (±SEM) of (a) absolute LF HRV and (b) absolute HF HRV in the Hypnosis condition (black circles) versus the Control condition (white circles). Light gray area indicates nap time, darker gray area within nap time indicates duration of the audio tape. **$p < 0.01$, $^{\#}p < 0.10$ for the Condition main effect of the linear mixed models analyses shown in the insets; n = 22.

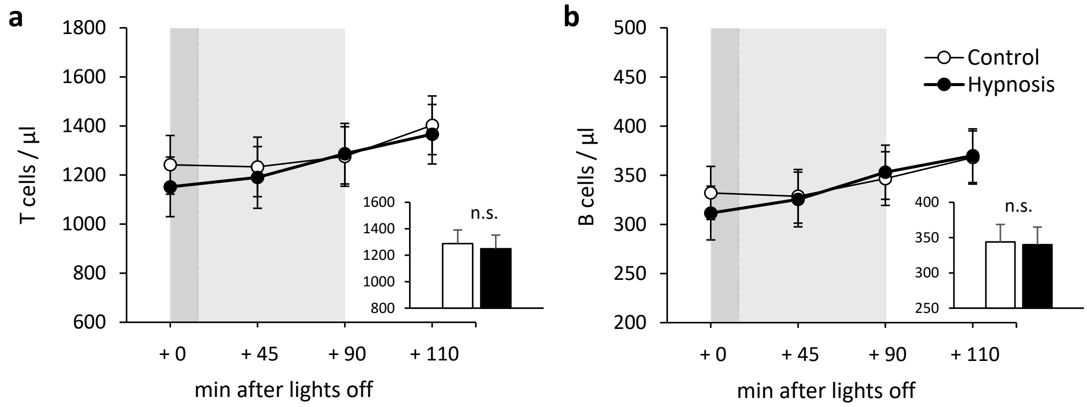

Supplementary Figure S3. Effects of hypnotic suggestions on numbers of circulating lymphocytes. Estimated marginal means (±SEM) of (a) T cell and (b) B cell numbers in blood in the Hypnosis condition (black circles) versus the Control condition (white circles). Light gray area indicates nap time, darker gray area within nap time indicates duration of the audio tape. The insets show the results of the Condition main effect of the linear mixed models analyses; n.s., not significant; n = 15.

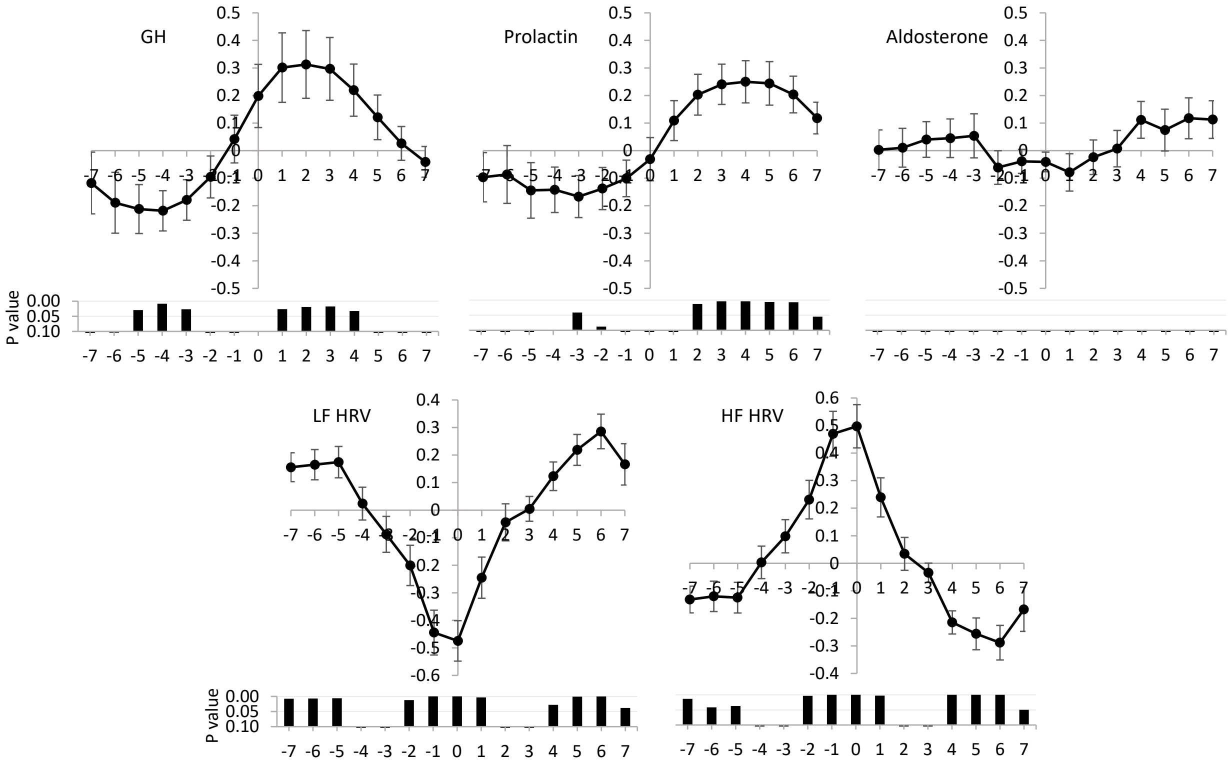

Supplementary Figure S4. Cross correlations for the temporal course of slow-wave sleep (SWS) and peripheral body parameters in the Hypnosis condition. Means (±SEM) of cross-correlation coefficients and respective P values for SWS and growth hormone (GH), prolactin, aldosterone, the relative low frequency (LF) power of heart-rate variability (HRV) and high frequency HRV (HF HRV), respectively, for lags -7 to +7. Each lag corresponds to a 5-min interval; n = 22.
