# Supplementary material for: Hypnotic enhancement of slow-wave sleep increases sleep-associated hormone secretion and reduces sympathetic predominance in healthy humans
Source: Commun Biol. 2022 Jul 26;5:747. doi: 10.1038/s42003-022-03643-y (PMC9325885; doi:10.1038/s42003-022-03643-y)
Supplement: Supplementary file 3 — Description of Additional Supplementary Files [file 42003_2022_3643_MOESM3_ESM.pdf]

Description of Additional Supplementary Files

File name: Supplementary Data 1
Description: Source data for Figures 1 – 3.
