# Supplementary material for: Hypnotic enhancement of slow-wave sleep increases sleep-associated hormone secretion and reduces sympathetic predominance in healthy humans
Source: Commun Biol. 2022 Jul 26;5:747. doi: 10.1038/s42003-022-03643-y (PMC9325885; doi:10.1038/s42003-022-03643-y)
Supplement: Supplementary file 5 — Reporting Summary [file 42003_2022_3643_MOESM5_ESM.pdf]

nature portfolio

Corresponding author(s): Luciana Besedovsky, Björn Rasch

Last updated by author(s): May 10, 2022

Reporting Summary

Nature Portfolio wishes to improve the reproducibility of the work that we publish. This form provides structure for consistency and transparency in reporting. For further information on Nature Portfolio policies, see our Editorial Policies and the Editorial Policy Checklist.

Statistics

For all statistical analyses, confirm that the following items are present in the figure legend, table legend, main text, or Methods section.

n/a	Confirmed	
☐	☒	The exact sample size (n) for each experimental group/condition, given as a discrete number and unit of measurement
☒	☐	A statement on whether measurements were taken from distinct samples or whether the same sample was measured repeatedly
☐	☒	The statistical test(s) used AND whether they are one- or two-sided<br>*Only common tests should be described solely by name; describe more complex techniques in the Methods section.*
☒	☐	A description of all covariates tested
☐	☒	A description of any assumptions or corrections, such as tests of normality and adjustment for multiple comparisons
☐	☒	A full description of the statistical parameters including central tendency (e.g. means) or other basic estimates (e.g. regression coefficient) AND variation (e.g. standard deviation) or associated estimates of uncertainty (e.g. confidence intervals)
☐	☒	For null hypothesis testing, the test statistic (e.g. F, t, r) with confidence intervals, effect sizes, degrees of freedom and P value noted<br>*Give P values as exact values whenever suitable.*
☒	☐	For Bayesian analysis, information on the choice of priors and Markov chain Monte Carlo settings
☒	☐	For hierarchical and complex designs, identification of the appropriate level for tests and full reporting of outcomes
☐	☒	Estimates of effect sizes (e.g. Cohen's d, Pearson's r), indicating how they were calculated

Our web collection on statistics for biologists contains articles on many of the points above.

Software and code

Policy information about availability of computer code

Data collection	N/A
Data analysis	Statistical analyses were performed with IBM SPSS statistics, version 26.

For manuscripts utilizing custom algorithms or software that are central to the research but not yet described in published literature, software must be made available to editors and reviewers. We strongly encourage code deposition in a community repository (e.g. GitHub). See the Nature Portfolio guidelines for submitting code & software for further information.

Data

Policy information about availability of data

All manuscripts must include a data availability statement. This statement should provide the following information, where applicable:

- Accession codes, unique identifiers, or web links for publicly available datasets
- A description of any restrictions on data availability
- For clinical datasets or third party data, please ensure that the statement adheres to our policy

Source data for Figures 1 – 3 can be found in Supplementary Data 1. All other data are available from the corresponding authors on reasonable request.

Field-specific reporting

Please select the one below that is the best fit for your research. If you are not sure, read the appropriate sections before making your selection.

☒ Life sciences ☐ Behavioural & social sciences ☐ Ecological, evolutionary & environmental sciences

For a reference copy of the document with all sections, see nature.com/documents/nr-reporting-summary-flat.pdf

Life sciences study design

All studies must disclose on these points even when the disclosure is negative.

Sample size	The sample size was calculated a priori using power analyses based on previous studies using hypnotic suggestions and pharmacological interventions to increase SWS.
Data exclusions	Eight Participants were excluded during the experiments because of problems with the intravenous catheter, 5 were excluded because of non-compliance (e.g., did not follow a regular sleep-wake schedule before experiments), 2 reported having a cold and took pain medication shortly before the experiment, one did not meet further inclusion criteria, and 5 dropped out for personal reasons.
Replication	N/A
Randomization	All participants participated in both experimental conditions according to a within-subjects design. The order of conditions was balanced.
Blinding	Participants did not know which audio tape (hypnosis or neutral text) would be presented before having the nap. Investigators were blinded during analysis.

Reporting for specific materials, systems and methods

We require information from authors about some types of materials, experimental systems and methods used in many studies. Here, indicate whether each material, system or method listed is relevant to your study. If you are not sure if a list item applies to your research, read the appropriate section before selecting a response.

Materials & experimental systems

n/a	Involved in the study	
☐	☒	Antibodies
☒	☐	Eukaryotic cell lines
☒	☐	Palaeontology and archaeology
☒	☐	Animals and other organisms
☐	☒	Human research participants
☒	☐	Clinical data
☒	☐	Dual use research of concern

Methods

n/a	Involved in the study	
☒	☐	ChIP-seq
☐	☒	Flow cytometry
☒	☐	MRI-based neuroimaging

Antibodies

Antibodies used	Alexa Fluor® 700 anti-human CD45 (clone HI30, catalog #: 304024), Brilliant Violet 510 anti-human CD3 (clone UCHT1, catalog #: 300448), and FITC anti-human CD19 (clone SJ25C1, catalog #: 363008) (Biolegend, San Diego, CA, USA)
Validation	All antibodies are validated for use in human samples as stated by the manufacturer.

Human research participants

Policy information about studies involving human research participants

Population characteristics	Healthy men, mean age 23.5 years ± 2.71 SD, mean body-mass index: 23.0 kg/m2 ± 1.95 SD
Recruitment	Participants were recruited by advertisements sent via the e-mail distribution list for members of the University of Tübingen.
Ethics oversight	The study was approved by the Ethics Committee of the University of Tübingen.

Note that full information on the approval of the study protocol must also be provided in the manuscript.

Flow Cytometry

Plots

Confirm that:

☐ The axis labels state the marker and fluorochrome used (e.g. CD4-FITC).

☐ The axis scales are clearly visible. Include numbers along axes only for bottom left plot of group (a 'group' is an analysis of identical markers).

☐ All plots are contour plots with outliers or pseudocolor plots.

☒ A numerical value for number of cells or percentage (with statistics) is provided.

Methodology

Sample preparation	50 µl of an undiluted blood sample were immunostained with anti-CD45, anti-CD3, and anti-CD19 antibodies (at final dilutions of 1:333, 1:333, and 1:50, respectively) in Trucount tubes (Biolegend, San Diego, CA, USA). After 15 min of incubation at room temperature, 0.9 ml of FACS lysing solution (BD Biosciences, San Jose, CA, USA) was added to lyse erythrocytes for 15 min. Samples were then mixed gently, and at least 100,000 CD45+ cells were acquired on a BD LSRFortessa Flow Cytometer (BD Biosciences).
Instrument	BD LSRFortessa Flow Cytometer
Software	DIVA software, BD Biosciences
Cell population abundance	100,000 CD45+ cells were acquired. Approximately 40% of the cells were lymphocytes.
Gating strategy	100.000 CD45+ cells were acquired. Doublets were excluded based on their FSC-H/FSC-A properties. Lymphocytes were first roughly gated based on their FSC/SSC properies. CD45+ cells were selected. CD3+CD19- cells within this gate were defined as T cells and CD3-CD19+ cells were defined as B cells. Because the gating strategy is very basic and the results of these flow cytometry analyses are not central to the present study, we do not show any contour plots but only included graphs showing the cell numbers at the various measurement time points in both experimental conditions.

☐ Tick this box to confirm that a figure exemplifying the gating strategy is provided in the Supplementary Information.
